# The Environmental Health Literacy of Italian General Population: The SPeRA Cross-Sectional Study

**DOI:** 10.3390/ijerph20054486

**Published:** 2023-03-02

**Authors:** Fabrizio Bert, Marta Gea, Christian Previti, Gregorio Massocco, Giuseppina Lo Moro, Giacomo Scaioli, Tiziana Schilirò, Roberta Siliquini

**Affiliations:** 1 Department of Public Health and Pediatrics, University of Turin, 10126 Turin, Italy; 2 Hygiene and Infection Control Unit, ASL TO3, 10098 Turin, Italy; 3 AOU City of Health and Science of Turin, 10126 Turin, Italy

**Keywords:** environmental health literacy, pro-environmental behaviours, environmental risk perception, pro-environmental attitudes, adult population, environmental burden of diseases

## Abstract

Environmental health literacy (EHL) includes knowledge of health effects due to environmental exposure and skills to protect health from environmental risks. This study investigated some aspects about EHL of the Italian adult population. Data were collected through questionnaires (*n =* 672) and analysed through multivariable logistic regression models. Results showed that participants with incomplete/insufficient self-perceived knowledge of health effects due to environmental risks verified less information about this topic (adjOR = 0.38 (CI95% 0.25–0.59)/0.09 (0.04–0.21); *p* < 0.001/<0.001), potentially spreading fake news. The self-perceived exposure to pollution was higher in participants living in towns than in rural areas (small, medium, big towns adjOR = 2.37 (1.41–3.97), 2.10 (1.11–3.96), 3.11 (1.53–6.31); *p* = 0.001, 0.022, 0.002) and lower in participants with incomplete/insufficient knowledge about pollution effects (adjOR = 0.54 (0.32–0.92)/0.30 (0.13–0.67); *p* = 0.022/0.004), confirming that knowledge is essential to achieve awareness. Since insufficient self-perceived knowledge of pollution effects was negatively associated with the adoption of pro-environmental behaviours (adjOR = 0.37 (0.15–0.90); *p* = 0.028), EHL was proven to be a virtuous behaviour promoter. Finally, a lack of institutional support, time and cost were identified as barriers to pro-environmental behaviours. This study provided useful data to design prevention programmes, underlined some barriers to pro-environmental behaviours and highlighted the need to promote attitudes and behaviours aimed at contrasting environmental pollution, thus protecting human health.

## 1. Introduction

Worldwide, almost a quarter of mortality is due to environmental causes. Indeed, in 2016, the World Health Organization estimated that globally 24% of all deaths and 28% of deaths in children aged less than five years are caused by environmental factors, corresponding to 13.7 million deaths and 1.6 million deaths in children less than five years old per year [1]. These deaths are attributable to many environmental risk factors, including chemical and biological pollution of air, water and soil, ultraviolet and ionising radiation, occupational hazards and inadequate working conditions, noise, electromagnetic fields, climate change, availability of drinking water and farming techniques [1]. Besides self-caused environmental risk factors such as smoking, environmental pollution is the main environmental cause of premature deaths. Indeed, in 2015 it was responsible for 16% of all deaths (corresponding to 9 million deaths) and, specifically, it caused 26% of deaths due to ischemic heart disease, 23% of deaths due to stroke, 51% of deaths due to chronic obstructive pulmonary disease and 43% of deaths due to lung cancer [2].

Since human health is tightly connected to the environment, there is increasing interest in the research area focused on environmental health, which is a branch of public health aimed at preventing human injury/illness and promoting human well-being by identifying, assessing and limiting exposure to environmental hazards and hazardous physical, chemical and biological agents [3]. Within this research area, a starting point for planning interventions to improve human health and well-being is represented by the study of environmental health literacy (EHL).

EHL is composed of skills and competencies that people need in order to seek, understand, evaluate and use environmental health information. These elements can be used to make informed choices, reduce health risks, improve life quality and protect the environment [4]. EHL is an emerging and constantly evolving concept that starts from an understanding of the link between exposure to environmental risk factors and health and encompasses a set of knowledge and skills that enables people to make decisions and choices in order to preserve their health. It is not just an educational process, but it is a public health tool that can increase the health literacy of individuals and communities, reducing the exposure to environmental risks and thereby preventing disease outbreaks [5]. This literacy is closely related to other literacies, including scientific, health and environmental literacies [4]. Moreover, it is also associated with numerous factors such as sources of environmental health information, attitudes and sociodemographic characteristics [6].

Internationally, many studies on EHL have been performed in order to investigate the literacy level of different communities regarding environmental risk factors, also considering information sources [4]. EHL has been assessed in relation to generic environmental risk factors [7,8,9] or in relation to specific risk factors such as lead [6], endocrine disruptors [10] and pesticides [11]. These studies were performed in different geographic areas such as the United States [6,8,9], Chile [12], China [13] and Taiwan [14]. However, to our knowledge, EHL and risk perception regarding environmental risk factors have only been investigated by one study performed in Italy [15,16]. This study was focused on Italian students from 15 universities, while there are no data on EHL and risk perception of the general Italian population.

Therefore, the aim of the SPeRA cross-sectional study was to investigate some aspects of EHL and risk perception of the Italian adult population regarding environmental risk factors, in particular toward environmental pollution. The aspects considered were information sources and trust in information regarding health effect induced by environmental pollution, risk perception of environmental pollution, importance of institutional and non-institutional subjects to control environmental risk and perceived importance and adoption of behaviours aimed at reducing environmental pollution. These aspects were evaluated considering the socio-demographic characteristics of the research participants.

## 2. Materials and Methods

### 2.1. Study Design

A national cross-sectional study was performed (between 1 November 2021 and 16 May 2022) through an online questionnaire distributed on the main social media platforms (Facebook, Twitter and WhatsApp). Participants were required to read and sign an informed consent form prior to questionnaire filling. The language of both the questionnaire and informed consent was Italian. Only people aged 18 years or older and resident in Italy were allowed to participate in filling out the questionnaire. Participation was voluntary and without compensation. Fully anonymised data were collected through the online service UniQuest (LimeSurvey) provided by the University of Turin (Italy). The study was conducted in accordance with the 1975 Declaration of Helsinki (revised in 2013), and the protocol was approved by the Ethics Committee of the University of Turin.

### 2.2. Questionnaires

The self-administered questionnaire was composed of thirty-three items divided into six sections. Section 1 was about socio-demographic information (gender, age, nationality, town of residence, education level, employment status and presence/absence of children) while Section 2 was focused on the information about health effects induced by environmental pollution. In Section 3, the perception of the health risk associated with environmental pollution was investigated. Section 4 looked at what the subjects considered to be important regarding environmental pollution. Finally, Section 5 explored participants’ adoption of behaviours aimed at reducing environmental pollution (pro-environmental behaviours) and attitudes/motivations toward adopting them. The questionnaire was constructed by the research team taking the validated questionnaire items from the studies [15,16] as a model.

### 2.3. Data Analysis and Statistical Analysis

Questionnaire answers were analysed using the STATA software (StataCorp LLC, College Station, TX, USA). Descriptive analyses were performed for all variables. Specifically, continuous variables were evaluated as the mean value and standard deviation, while categorical variables were expressed as percentage values. The percentage values were calculated excluding the answers “I don’t know” and “I don’t use this information source” (for some items, these answers were selected by a high number of participants). Multivariable logistic regression models adjusted for age and gender were run to assess how the variables analysed in the questionnaire were associated with five binary outcomes.

The five considered outcomes were as follows:

1. Verifying information about the health effects of pollution. The participants that answered “Often” and “Always” to the question “How often do you verify information about health effects due to environmental pollution before believing or disclosing it?” were considered to verify information; in contrast, the participants that answered “Never”, “Sporadically” and “Sometimes” were considered not to verify information. The choice was made considering the frequency of this behaviour (i.e., verifying information).
2. Self-perceived exposure to pollution. A positive self-perceived exposure to pollution was considered for the participants that answered “Often” and “Always” to the question “How often do you feel exposed to environmental pollution?”, while an absence of self-perceived exposure was considered for the participants that answered “Never”, “Sporadically” and “Sometimes”. The choice was made considering the frequency of self-perceived exposure to pollution.
3. Estimation of burden of disease from environmental causes. A high estimation of burden was considered for the participants who answered “41–60%” and “>60%” to the question “What is the percentage of diseases due to environmental pollution in the world?” This cut-off was selected considering that the burden of environmental diseases reported by WHO is equal to 24% [1].
4. Perceived importance of pro-environmental behaviours. The question considered for this outcome was “How important are the following behaviours to reduce environmental pollution?” A score of 1 or 0 was given to each pro-environmental behaviour depending on the given answer (I don’t know = 0, Not important = 0, Little importance = 0, Moderately important = 0, Very important = 1, Extremely important = 1). The behaviours “Reducing alcohol consumption”, “Online shopping” and “Doing physical activity” were not considered to be pro-environmental behaviours; therefore, they were scored in an opposite sense (I don’t know = 1, Not important = 1, Little importance = 1, Moderately important = 1, Very important = 0, Extremely important = 0). The sum of the scores of each behaviour resulted in a cumulative score variable between 0 and 16. A score < 12 was considered as medium–low perceived importance of pro-environmental behaviours, while a score ≥ 12 was considered as high perceived importance of these behaviours. Since most of the participants achieved a high cumulative score, the cut-off of 12 instead of 8 was selected in order to divide the sample into two comparable groups.
5. Adoption of pro-environmental behaviours. The question considered for this outcome was “How often do you adopt the following behaviours?” A score of 1 or 0 was given to each pro-environmental behaviour depending on the given answer (I don’t know = 0, Never = 0, Sporadically = 0, Sometimes = 0, Often = 1, Always = 1). The behaviours “Reduce alcohol consumption”, “Shop online” and “Do physical activity” were excluded, as they were not considered to be pro-environmental behaviours. The sum of the scores of each pro-environmental behaviour resulted in a cumulative score variable between 0 and 13. A score < 7 was considered to be a low frequency of adopting pro-environmental behaviours, while a score ≥ 7 was considered to be a high frequency of adopting these behaviours. This score was selected in order to divide the sample into two comparable groups.

Within the models, variables related to gender, age, size of town where participants live, education level, presence/absence of children, employment status, opinion on information about health effects induced by pollution, self-perceived knowledge about health effects due to pollution, self-perceived exposure to pollution and perceived importance of pro-environmental behaviours were included. The results of the logistic regression models were expressed as adjusted odds ratios (adjORs) and 95% confidence intervals (CI95%). The goodness-of-fit of the models was evaluated using log likelihood chi-square and Hosmer and Lemeshow’s goodness-of-fit test.

## 3. Results

### 3.1. Descriptive Analysis

#### 3.1.1. Socio-Demographic Information (Table S1, Supplementary Materials)

The study population was composed of 672 participants aged 46.53 ± 14.53 years and people of mainly Italian nationality (97.99%). Regarding gender, 71.13% of the participants were female, 28.13% were male and 0.74% were non-binary.

#### 3.1.2. Information on Health Effects Induced by Environmental Pollution (Table S2, Supplementary Materials)

Information regarding health effects induced by environmental pollution was generally considered to be true, but incomplete (73.66%), and more than half of the participants stated that they often (34.84%) or always (20.58%) check its truthfulness before believing or divulging it. The most used source of information on the health effects induced by environmental pollution was the Internet (70.43%), followed by TV (42.52%), newspapers and magazines (24.09%) and social media (20.43%).

Although the Internet was the main information source, 57.69% of the participants considered this source to be partially or not reliable. In addition, TV and social media were also considered to be partially or not reliable by many participants (by 59.42% and 84.24% of the participants, respectively). Newspapers and magazines were considered to be more reliable than the Internet, TV and social media, as only 43.30% of participants considered them to be partially or not reliable.

#### 3.1.3. Risk Perception of Environmental Pollution (Table S3, Supplementary Materials)

A high percentage of participants stated that they often (45.32%) or always (29.78%) felt exposed to environmental pollution. They started to think about the health effects induced by environmental pollution at 26.54 years (±11.20 years), and most of them (61.86%) considered their knowledge about this topic to be incomplete. Regarding the estimate of the percentage of diseases due to it, more than half of the participants stated that it causes more than 40% of all diseases (38.17% estimated 41–60% of all diseases, while 24.95% estimated >60% of all diseases).

Most of the participants declared that environmental pollution induces both short-term and long-term effects on health (70.13%), while half of the participants thought that it affects all people in the same way independently of age (53.01%).

The results showed that environmental pollution was believed to have a very important or an extremely important role in the development of respiratory diseases (by 92.02% of participants), cancers (by 85.85% of participants) and congenital/neonatal malformations (by 72.09% of participants). On the contrary, a smaller percentage of participants considered pollution to be important in the development of cardiovascular diseases, neurological diseases and dementia, infectious diseases, psychiatric disorders, gastrointestinal diseases and disorders of sense organs.

Industrial pollution, groundwater pollution, surface water pollution, chemicals in food and drinking water and vehicular traffic were considered to have a very important or an extremely important negative effect on human health by many participants (92.43%, 92.02%, 90.85%, 87.82% and 86.73%, respectively). On the contrary, other factors, such as climate change, nuclear power plants, home heating, air quality, traffic noise, landfills, incinerators and biological contamination of food/water were considered to have a very important or an extremely important negative effect on human health by a lower percentage of participants. Moreover, among the lifestyle exposure factors, cigarette smoke and sunlight exposure without sunscreen were considered by the majority of participants to have a very important or an extremely important negative effect on human health (92.18% and 72.73%, respectively).

#### 3.1.4. Importance of Institutional and Non-Institutional Subjects to Control the Risk Due to Environmental Pollution (Table S4, Supplementary Materials)

According to most participants, the environmental pollution was uncontrollable or only partially controllable (56.55%), and also the human health risk due to the environmental pollution was considered to be uncontrollable or only partially controllable by many participants (65.35%).

The citizens were believed to be very important or extremely important in controlling this risk by 88.43% of participants. However, 90.36% of the participants stated that the risk has not been resolved or has only been partially resolved through public awareness.

The government, the Environmental Ministry and the environmental protection agencies were also considered to be very or extremely important in controlling health risk due to environmental pollution by a high percentage of participants (85.24%, 83.30% and 82.30%, respectively). However, 94.75% of the participants stated that the actions taken by these institutions were not or were only partially effective in resolving this problem.

#### 3.1.5. Pro-Environmental Behaviours: Perceived Importance and Adoption (Tables S5–S8, Supplementary Materials)

Purchasing ecolabel products and reducing meat consumption were considered as being very or extremely important pro-environmental behaviours by a low percentage of the participants (52.55%, 56.75%). On the contrary, separate waste collection, using low-impact products and reducing energy consumption were considered to be very or extremely important pro-environmental behaviours by most participants (90.91%, 90.69% and 88.24%); these were also the most frequently adopted behaviours since 95.98%, 77.13% and 76.63% of the participants often or always adopts them.

Moreover, using green fuels and sustainable transport were considered to be very/extremely important pro-environmental behaviours by 83.82% and 81.11% of participants; however, only 34.88% and 29.91% of the participants often or always adopts them. Cost and lack of institutional support were considered the main barriers to using green fuels, while lack of time and institutional support were considered the main barriers to using sustainable transport. Finally, using public transport was considered to be a very or extremely important pro-environmental behaviour by 73.43% of participants, as well as the purchasing of zero-mile products (considered a very or extremely important behaviour by 74.54%). However, only 29.29% and 38.50% of participants often or always adopts them because of lack of institutional support/lack of time and cost/lack of time, respectively.

The participants declared that the lack of institutional support was the main barrier to separate waste collection and the use of low-impact products. Cost was identified as being the main barrier to purchasing ecolabel products, to reducing energy consumption and to sustainable tourism, while time was identified as the main barrier to planting trees. Finally, the participants declared that the lack of support from family members was the main barrier to reducing home heating during winter, air conditioning during summer and meat consumption.

Regarding the motivations to adopt pro-environmental behaviours, a high percentage of participants considered as very or extremely important motivations regarding the protection of their health (95.79%), the protection of the environment (93.32%) and the protection of other people’s health (91.84%). Instead, a smaller percentage of participants considered as very or extremely important motivations regarding economic benefit (43.07%) and approval by other people (22.03%).

### 3.2. Multivariable Logistic Regression Models

#### 3.2.1. Verifying Information about the Health Effects of Pollution (Table 1)

The results showed that the participants with incomplete or insufficient self-perceived knowledge of health effects due to environmental pollution verify the information about the health effects of pollution less than the participants with sufficient self-perceived knowledge (incomplete knowledge: adjOR = 0.38 (CI95% 0.25–0.59), *p* < 0.001; insufficient knowledge: adjOR = 0.09 (CI95% 0.04–0.21), *p* < 0.001).

**Table 1:** Multivariable logistic regression model on the first outcome: verifying information about the health effects of pollution.

Item	Answer	adjOR (CI95%)	*p*-Value
Gender	Male	1	-
	Female	1.23 (0.81–1.86)	0.325
Age	Years	1.02 (0.99–1.04)	0.062
Town of residence	Very small town	1	-
	Small town (≤50,000 inhabitants)	0.80 (0.50–1.29)	0.368
	Medium town (50,001–250,000 inhabitants)	0.67 (0.38–1.16)	0.153
	Big town (>250,000 inhabitants)	0.59 (0.33–1.08)	0.086
Education level	Middle school diploma or less	1	-
	High school diploma	0.92 (0.50–1.69)	0.790
	Bachelor’s degree	1.47 (0.70–3.06)	0.307
	Master’s degree	1.72 (0.87–3.40)	0.118
	Doctoral degree	1.52 (0.63–3.69)	0.351
Children	No	1	-
	Yes	0.68 (0.44–1.06)	0.094
Employment status	Worker	1	-
	Unemployed	1.48 (0.80–2.72)	0.209
	Student	0.71 (0.25–2.05)	0.531
	Retired	0.78 (0.40–1.52)	0.469
	Working student	3.25 (0.92–11.50)	0.068
Opinion on information about health effects induced by environmental pollution	True and complete	1	-
	True but incomplete	0.78 (0.45–1.34)	0.368
	Not true and incomplete	0.95 (0.44–2.08)	0.905
	I don’t know	1.22 (0.51–2.93)	0.652
Self-perceived knowledge about health effects due to environmental pollution	Sufficient	1	-
	Incomplete	0.38 (0.25–0.59)	<0.001
	Insufficient	0.09 (0.04–0.21)	<0.001

Goodness-of-fit tests: log likelihood chi-square = 68.53, df = 19; *p* < 0.001; Hosmer and Lemeshow’s, *p* = 0.646.

#### 3.2.2. Self-Perceived Exposure to Pollution (Table 2)

The results showed that females had a higher self-perceived exposure to pollution than males (females: adjOR = 2.40 (CI95% 1.52–3.80), *p* < 0.001) and elders had higher self-perceived exposure to pollution than young participants (years: adjOR = 1.02 (CI95% 1.01–1.05), *p* = 0.043).

Moreover, the perceived exposure to pollution was lower in retired participants than in workers (retired participants: adjOR = 0.35 (CI95% 0.17–0.76), *p* = 0.007) and higher in participants that live in small, medium and big towns than in those that live in very small towns (small towns: adjOR = 2.37 (CI95% 1.41–3.97), *p* = 0.001; medium towns: adjOR = 2.10 (CI95% 1.11–3.96), *p* = 0.022; big towns: adjOR = 3.11 (CI95% 1.53–6.31), *p* = 0.002).

Finally, the participants with incomplete or insufficient self-perceived knowledge of health effects due to environmental pollution considered themselves as being less exposed to environmental pollution than the participants with sufficient self-perceived knowledge (incomplete knowledge: adjOR = 0.54 (CI95% 0.32–0.92), *p* = 0.022; insufficient knowledge: adjOR = 0.30 (CI95% 0.13–0.67), *p* = 0.004).

**Table 2:** Multivariable logistic regression model on the second outcome: self-perceived exposure to pollution.

Item	Answer	adjOR (CI95%)	*p*-Value
Gender	Male	1	-
	Female	2.40 (1.52–3.80)	<0.001
Age	Years	1.02 (1.01–1.05)	0.043
Town of residence	Very small town	1	-
	Small town (≤50,000 inhabitants)	2.37 (1.41–3.97)	0.001
	Medium town (50,001–250,000 inhabitants)	2.10 (1.11–3.96)	0.022
	Big town (>250,000 inhabitants)	3.11 (1.53–6.31)	0.002
Education level	Middle school diploma or less	1	-
	High school diploma	1.10 (0.56–2.15)	0.791
	Bachelor’s degree	2.03 (0.86–4.78)	0.106
	Master’s degree	1.35 (0.63–2.93)	0.442
	Doctoral degree	1.08 (0.39–2.96)	0.881
Children	No	1	-
	Yes	1.28 (0.76–2.15)	0.363
Employment status	Worker	1	-
	Unemployed	1.40 (0.64–3.05)	0.402
	Student	1.05 (0.35–3.11)	0.930
	Retired	0.35 (0.17–0.76)	0.007
	Working student	5.93 (0.73–48.46)	0.097
Opinion on information about health effects induced by environmental pollution	True and complete	1	-
	True but incomplete	1.50 (0.81–2.80)	0.200
	Not true and incomplete	1.50 (0.61–3.71)	0.376
	I don’t know	0.99 (0.39–2.53)	0.981
Self-perceived knowledge about health effects due to environmental pollution	Sufficient	1	-
	Incomplete	0.54 (0.32–0.92)	0.022
	Insufficient	0.30 (0.13–0.67)	0.004

Goodness-of-fit tests: log likelihood chi-square = 60.99, df = 20; *p* < 0.001; Hosmer and Lemeshow’s, *p* = 0.646.

#### 3.2.3. Estimation of Burden of Disease from Environmental Causes (Table 3)

The burden of disease from environmental causes was considered to be higher by females than by males (females: adjOR = 2.73 (CI95% 1.73–4.32), *p* < 0.001) and lower by the participants with a high education level (high school diploma, Bachelor’s degree, a Master’s degree and a doctoral degree) than by the participants with a middle school diploma or less (high school diploma: adjOR = 0.33 (CI95% 0.13–0.82), *p* = 0.017; Bachelor’s degree: adjOR = 0.20 (CI95% 0.08–0.55), *p* = 0.002; Master’s degree: adjOR = 0.16 (CI95% 0.06–0.42), *p* < 0.001; doctoral degree: adjOR = 0.17 (CI95% 0.05–0.52), *p* = 0.002). Moreover, the estimation of burden was considered to be higher by the participants that declared to be exposed to pollution than by the others (positive self-perceived exposure to pollution: adjOR = 1.94 (CI95% 1.17–3.20), *p* = 0.010).

**Table 3:** Multivariable logistic regression model on the third outcome: estimation of burden of disease from environmental causes.

Item	Answer	adjOR (CI95%)	*p*-Value
Gender	Male	1	-
	Female	2.73 (1.73–4.32)	<0.001
Age	Years	1.02 (0.99–1.04)	0.145
Town of residence	Very small town	1	-
	Small town (≤50,000 inhabitants)	0.97 (0.56–1.67)	0.908
	Medium town (50,001–250,000 inhabitants)	1.16 (0.60–2.27)	0.659
	Big town (>250,000 inhabitants)	0.81 (0.42–1.57)	0.533
Education level	Middle school diploma or less	1	-
	High school diploma	0.33 (0.13–0.82)	0.017
	Bachelor’s degree	0.20 (0.08–0.55)	0.002
	Master’s degree	0.16 (0.06–0.42)	<0.001
	Doctoral degree	0.17 (0.05–0.52)	0.002
Children	No	1	-
	Yes	1.19 (0.72–1.96)	0.498
Employment status	Worker	1	-
	Unemployed	1.62 (0.71–3.69)	0.250
	Student	0.41 (0.13–1.29)	0.126
	Retired	0.75 (0.34–1.63)	0.464
	Working student	0.60 (1.19–1.87)	0.378
Self-perceived knowledge about health effects due to environmental pollution	Sufficient	1	-
	Incomplete	0.79 (0.49–1.29)	0.347
	Insufficient	0.47 (0.21–1.08)	0.074
Self-perceived exposure to environmental pollution	No	1	-
	Yes	1.94 (1.17–3.20)	0.010

Goodness-of-fit tests: log likelihood chi-square = 79.09, df = 17; *p* < 0.001; Hosmer and Lemeshow’s, *p* = 0.313.

#### 3.2.4. Perceived Importance of Pro-Environmental Behaviours (Table 4)

The fourth multivariable logistic regression model showed that females, compared to males, had a higher perceived importance of pro-environmental behaviours (females: adjOR = 1.98 (CI95% 1.26–3.10), *p* = 0.003). A higher perceived importance was also found in participants with a Bachelor’s degree, a Master’s degree and a doctoral degree, with respect to participants with a middle school diploma or less (Bachelor’s degree: adjOR = 2.46 (CI95% 1.09–5.55), *p* = 0.030; Master’s degree: adjOR = 2.54 (CI95% 1.22–5.30), *p* = 0.013; doctoral degree: adjOR = 4.19 (CI95% 1.58–11.11), *p* = 0.004). Finally, the perceived importance of pro-environmental behaviours was considered to be higher by the participants that declared to be exposed to pollution than by the others (positive self-perceived exposure to pollution: adjOR = 2.10 (CI95% 1.30–3.38), *p* = 0.002).

**Table 4:** Multivariable logistic regression model on the fourth outcome: perceived importance of pro-environmental behaviours.

Item	Answer	adjOR (CI95%)	*p*-Value
Gender	Male	1	-
	Female	1.98 (1.26–3.10)	0.003
Age	Years	0.99 (0.97–1.02)	0.669
Town of residence	Very small town	1	-
	Small town (≤50,000 inhabitants)	0.90 (0.54–1.51)	0.693
	Medium town (50,001–250,000 inhabitants)	0.83 (0.45–1.54)	0.552
	Big town (>250,000 inhabitants)	0.82 (0.43–1.56)	0.546
Education level	Middle school diploma or less	1	-
	High school diploma	1.86 (0.96–3.63)	0.068
	Bachelor’s degree	2.46 (1.09–5.55)	0.030
	Master’s degree	2.54 (1.22–5.30)	0.013
	Doctoral degree	4.19 (1.58–11.11)	0.004
Children	No	1	-
	Yes	1.35 (0.83–2.18)	0.222
Employment status	Worker	1	-
	Unemployed	0.99 (0.50–1.93)	0.967
	Student	0.53 (0.17–1.65)	0.276
	Retired	1.39 (0.67–2.88)	0.375
	Working student	1.36 (0.37–5.02)	0.645
Self-perceived knowledge about health effects due to environmental pollution	Sufficient	1	-
	Incomplete	0.68 (0.43–1.07)	0.094
	Insufficient	0.77 (0.35–1.68)	0.508
Self-perceived exposure to environmental pollution	No	1	-
	Yes	2.10 (1.30–3.38)	0.002

Goodness-of-fit tests: log likelihood chi-square = 42.72, df = 18; *p* < 0.001; Hosmer and Lemeshow’s, *p* = 0.178.

#### 3.2.5. Adoption of Pro-Environmental Behaviours (Table 5)

The adoption of pro-environmental behaviours was higher in elder participants than in younger ones (years: adjOR = 1.04 (CI95% 1.02–1.07), *p* < 0.001), while it was lower in participants with children than in the others (children: adjOR = 0.46 (CI95% 0.28–0.77), *p* = 0.003). These behaviours were less adopted by participants with a high school diploma with respect to participants with a middle school diploma or less (high school diploma: adjOR = 0.43 (CI95% 0.21–0.91), *p* = 0.027). Moreover, they were also less adopted by participants with an insufficient self-perceived knowledge of health effects due to environmental pollution than with sufficient knowledge (insufficient knowledge: adjOR = 0.37 (CI95% 0.15–0.90), *p* = 0.028). Finally, the adoption of pro-environmental behaviour was higher in participants that considered these behaviours to be important (high importance: adjOR = 4.91 (CI95% 3.13–7.70), *p* < 0.001).

**Table 5:** Multivariable logistic regression model on the fifth outcome: adoption of pro-environmental behaviours.

Item	Answer	adjOR (CI95%)	*p*-Value
Gender	Male	1	-
	Female	1.60 (0.97–2.65)	0.067
Age	Years	1.04 (1.02–1.07)	<0.001
Town of residence	Very small town	1	-
	Small town (≤50,000 inhabitants)	1.15 (0.66–2.00)	0.618
	Medium town (50,001–250,000 inhabitants)	0.88 (0.45–1.72)	0.719
	Big town (>250,000 inhabitants)	1.13 (0.57–2.23)	0.730
Education level	Middle school diploma or less	1	-
	High school diploma	0.43 (0.21–0.91)	0.027
	Bachelor’s degree	0.74 (0.31–1.79)	0.505
	Master’s degree	0.69 (0.31–1.55)	0.370
	Doctoral degree	0.84 (0.30–2.36)	0.744
Children	No	1	-
	Yes	0.46 (0.28–0.77)	0.003
Employment status	Worker	1	-
	Unemployed	1.77 (0.85–3.69)	0.127
	Student	0.74 (0.20–2.77)	0.661
	Retired	1.04 (0.47–2.28)	0.929
	Working student	1.02 (0.28–3.70)	0.981
Self-perceived knowledge about health effects due to environmental pollution	Sufficient	1	-
	Incomplete	0.69 (0.43–1.12)	0.136
	Insufficient	0.37 (0.15–0.90)	0.028
Self-perceived exposure to environmental pollution	No	1	-
	Yes	1.31 (0.77–2.23)	0.322
Perceived importance of pro-environmental behaviours	Low-medium	1	-
	High	4.91 (3.13–7.70)	<0.001

Goodness-of-fit tests: log likelihood chi-square = 104.07, df = 19; *p* < 0.001; Hosmer and Lemeshow’s, *p* = 0.538.

## 4. Discussion

Environmental pollution, together with resource exploitation, is one of the greatest challenges of the contemporary world and will have an impact on future generations [17]. Industrial but also domestic activities significantly contribute to environmental pollution that threatens both human and global health. Therefore, the awareness and commitment of citizens to deal with this issue appear to be crucial.

Since knowledge and risk perception of the health effects due to environmental pollution may promote the adoption of attitudes and behaviours aimed at protecting the environment [18], the aim of this study was to investigate the EHL of the Italian general population, on which exhaustive data have not been reported in the literature.

The most used sources of information about the health effects of environmental pollution were the Internet and TV, and the obtained information was considered to be true but incomplete by a high percentage of the participants (73.66%). Indeed, the trust in information sources was quite low, since the Internet was considered to be partially or not reliable by 57.69% of participants, TV was considered by 59.42%, and even social media was considered by 84.24%. Similar data were also found by [12,15]. Indeed, the data of these studies highlighted a dissatisfaction in the information regarding the health effects of pollution. It is interesting to notice that, in the study [15], a greater use of social media as an information source was reported with respect to the present study; this is probably due to the lower age of the participants in the previous study (21 ± 4.3 years) compared to the present one (46.53 ± 14.53 years).

More than half of the participants stated that they often or always check information truthfulness before believing or divulging it; this could be due to a lack of trust in information sources. Interestingly, the multivariable logistic regression model showed that participants with incomplete or insufficient self-perceived knowledge of health effects due to environmental pollution verify the information less than participants with sufficient self-perceived knowledge (incomplete knowledge: adjOR = 0.38 (CI95% 0.25–0.59), *p* < 0.001; insufficient knowledge: adjOR = 0.09 (CI95% 0.04–0.21), *p* < 0.001). The lack of information checking combined with the lack of knowledge about the health effects of pollution could be a critical issue as they could lead to the dissemination of incomplete or untrue information, i.e., fake news. This news undermines the truthfulness of a topic, omitting or adding elements [19], and can undermine programmes, campaigns and initiatives aimed at raising awareness or educating the population [20]; thus, it can also hamper EHL.

A high percentage of the study participants stated that they felt exposed to environmental pollution. The self-perceived exposure to pollution was higher in those of female versus male gender and was correlated with age (females: adjOR = 2.40 (CI95% 1.52–3.80), *p* < 0.001; years: adjOR = 1.02 (CI95% 1.01–1.05), *p* = 0.043). This relationship between socio-demographic characteristics and risk perception is in accordance with the study [9] performed in the United States in 2019, in which cultural and socio-political dynamics were found to be associated with a different concern about environmental health risks. Furthermore, in the present study, the self-perceived exposure to pollution was higher in the participants living in small, medium and big towns than in those living in very small towns (small towns: adjOR = 2.37 (CI95% 1.41–3.97), *p* = 0.001; medium towns: adjOR = 2.10 (CI95% 1.11–3.96), *p* = 0.022; big towns: adjOR = 3.11 (CI95% 1.53–6.31), *p* = 0.002). This result could be explained considering that very small towns are generally located in rural areas where pollution is less evident than in urban areas; moreover, rural areas are generally considered to be environments not affected by pollution. The results also showed that self-perceived exposure to pollution is lower for the participants that have little/incomplete self-perceived knowledge of pollution health effects (incomplete knowledge: adjOR = 0.54 (CI95% 0.32–0.92), *p* = 0.022; insufficient knowledge: adjOR = 0.30 (CI95% 0.13–0.67), *p* = 0.004), confirming that the knowledge and possession of complete and truthful information are essential to achieve awareness. On the contrary, a lack of knowledge of the pollution health effects could lead to underestimating the different exposure methods and the different exposure contexts in which pollution can affect human health.

Regarding the estimation of the percentage of diseases due to environmental pollution, more than half of the participants (63.12%) stated that it causes more than 40% of all diseases, overestimating the burden of diseases as reported by WHO (equal to 24%) [1]. This is in accordance with the study [15], in which 82% of the participants (Italian students) overestimated the burden of diseases caused by environmental risk factors. [15] attributed the overestimation mainly to a lack of knowledge; this hypothesis is consistent with the present results. Indeed, in the present study, the overestimation was lower in participants with a higher education level (high school diploma: adjOR = 0.33 (CI95% 0.13–0.82), *p* = 0.017; Bachelor’s degree: adjOR = 0.20 (CI95% 0.08–0.55), *p* = 0.002; Master’s degree: adjOR = 0.16 (CI95% 0.06–0.42), *p* < 0.001; doctoral degree: adjOR = 0.17 (CI95% 0.05–0.52), *p* = 0.002). Finally, the overestimation was higher in females and in participants with a higher self-perceived exposure to pollution (females: adjOR = 2.73 (CI95% 1.73–4.32), *p* < 0.001; positive self-perceived exposure to pollution: adjOR = 1.94 (CI95% 1.17–3.20), *p* = 0.010).

Most of the participants considered separate waste collection, the use of low-impact products and energy consumption reduction to be the most important pro-environmental behaviours; these behaviours were also the most frequently adopted. This result is in accordance with the study [21], which found that the recycling of solid waste was considered to be important by Iranian students.

A higher perceived importance of pro-environmental behaviours was found in the participants with a higher self-perceived exposure to pollution (positive self-perceived exposure to pollution: adjOR = 2.10 (CI95% 1.30–3.38), *p* = 0.002); in addition, the adoption of pro-environmental behaviours was higher in the participants that considered them as being important (high importance: adjOR = 4.91 (CI95% 3.13–7.70), *p* < 0.001). Therefore, the perception of being exposed to pollution seems to be linked to an increased consideration of pro-environmental behaviours, which in turn promotes the adoption of pro-environmental behaviours. This connection was also confirmed by the results about motivations to adopt pro-environmental behaviour; indeed, the most important motivation was the protection of one’s own health.

The perceived importance of pro-environmental behaviour was also higher in the participants with a higher educational level, confirming that education is an important tool to protect the environment and human health (Bachelor’s degree: adjOR = 2.46 (CI95% 1.09–5.55), *p* = 0.030; Master’s degree: adjOR = 2.54 (CI95% 1.22–5.30), *p* = 0.013; doctoral degree: adjOR = 4.19 (CI95% 1.58–11.11), *p* = 0.004). It is interesting to notice that females, compared to males, had a higher perceived importance of pro-environmental behaviours and also a higher self-perceived exposure to pollution (females: adjOR = 1.98 (CI95% 1.26–3.10), *p* = 0.003 and adjOR = 2.40 (CI95% 1.52–3.80), *p* < 0.001, respectively). However, no statistically significant difference was found in the adoption of pro-environmental behaviours between females and males. This unexpected result could be due to barriers that may affect both genders similarly, such as cost, lack of time and institutional support.

Moreover, the adoption of pro-environmental behaviours was higher in older people and lower in participants with children (years: adjOR = 1.04 (CI95% 1.02–1.07), *p* < 0.001; children: adjOR = 0.46 (CI95% 0.28–0.77), *p* = 0.003); this could be explained considering that some barriers (e.g., lack of time, lack of support from family members and cost) could affect younger participants and parents more.

Finally, a negative association was found between the insufficient self-perceived knowledge of health effects due to environmental pollution and the adoption of pro-environmental behaviours (insufficient knowledge: adjOR = 0.37 (CI95% 0.15–0.90), *p* = 0.028), confirming the role of EHL in the reduction of environmental pollution and the related health effects.

According to the participants, citizens have a core role in limiting pollution and its related health effects, denoting a deep sense of responsibility. This sense of responsibility could favour the adoption of pro-environmental behaviours. According to the findings of the study [22], indeed, the assumption of responsibility has a direct influence on pro-environmental behaviours. The results showed that after citizens, the most important subjects in controlling the health risk due to environmental pollution are the government, the Environmental Ministry and the environmental protection agencies. However, the participants stated that neither public awareness nor institutions are effective in improving environmental pollution, suggesting a distrust in the actual possibility of controlling environmental pollution. This result is in accordance with the study [23]; indeed, many participants of this previous study declared that they did not believe that federal/local agencies were adequately protecting their health regarding environmental health risks. This result highlights the need to improve the knowledge about environmental health risks; indeed, this improvement can be used to promote a more optimistic view of the potential to mitigate, reduce or eliminate environmental exposures [5].

This is the first study about EHL and environmental pollution that was carried out in Italy on the general population. However, it was affected by some limitations. The COVID-19 pandemic, particularly in the first phase, required extensive containment measures (e.g., lockdown), and severely conditioned the possibility of pursuing this type of study using traditional diffusion methods. In compliance with epidemiological prevention measures, the research activity was conducted through online platforms, which could guarantee a suitable level of diffusion of the questionnaire. The use of dedicated software (UniQuest) for the collection and storage of information ensured faster and more effective data management and better respect for the participants’ privacy. On the other hand, the use of technological platforms may have limited access to the questionnaire to some groups of the Italian population (e.g., the elderly and people without access to the Internet). According to the most recent data (2021), 23.8% of Italian citizens aged 18 years or older declared that they never use the Internet (survey on 49,211 people, Italian Statistical Institute—ISTAT http://dati.istat.it/Index.aspx (accessed on 22 February 2023)). Furthermore, since the participant recruitment process was opportunistic, there may have been selection bias (e.g., people concerned with pollution issues may have been more prone to participate in the study). However, the population recruited in the present study appears to be quite representative of the Italian general population. Indeed, the mean age of the study participants (46.53 ± 14.53 years, participants aged 18 years or more) is similar to the mean age of Italian people aged 18 years or more (52.46 ± 18.90 years; data of the 1st January 2022; ISTAT http://dati.istat.it/Index.aspx (accessed on 27 February 2023)). Moreover, the employment status of the participants is also in agreement with the Italian data. Indeed, the employment rate in Italy is equal to 60.5% (data of December 2022; ISTAT http://dati.istat.it/Index.aspx (accessed on 27 February 2023)), and it is similar to the percentage of workers included in the present study (68.60%). Moreover, the Italian unemployment rate is equal to 7.8% (data of December 2022; ISTAT http://dati.istat.it/Index.aspx (accessed on 27 February 2023)) and is similar to the percentage of unemployed participants (10.74%). On the other hand, the prevalence of women filling out the questionnaire was higher (71.13%) than the prevalence of female subjects in Italy (data of December 2022; ISTAT http://dati.istat.it/Index.aspx (accessed on 27 February 2023)), in accordance with the results showing that male participants may have lower response rates [24]. Furthermore, the level of education declared by the participants was higher than that of the general Italian population (present study: middle school diploma or less = 11.71%, high school diploma = 43.58%, Bachelor’s/Master’s or doctoral degree = 44.71%; Italian data: middle school diploma or less = 42%, high school diploma = 41%, Bachelor’s/Master’s or doctoral degree = 17%; 2020 data, Italian population aged more than 20 years; ISTAT http://dati.istat.it/Index.aspx (accessed on 27 February 2023)). These aspects may influence the generalizability of the results of the present study.

## 5. Conclusions

This study confirmed that knowledge about pollution and its effects is important to raise risk perception and to favour the adoption of pro-environmental behaviours and attitudes. The study was also an opportunity to raise awareness among the population on environmental health, and the collected data could be useful to design public health projects/initiatives aimed at reducing and preventing exposure to environmental risk factors.

Since environmental pollution plays an important role in determining human health and EHL is strictly linked to environmental health effects, future studies are needed to collect more data on these topics in Italy and in all countries. These data will be useful to carry out targeted and functional education and prevention programmes that should be designed taking into account the level of knowledge of the target population and also its socio-demographic characteristics [25].

Moreover, this study highlighted the importance of barriers to pro-environmental behaviours; indeed, as reported by [16], these barriers could result in the non-adoption of pro-environmental behaviours even if they are perceived as being important. These barriers should be reduced as much as possible in order to protect the environment, and thus human health, from a one-health perspective.

## Data Availability

The data presented in this study are available upon reasonable request from the corresponding author.

## Supplementary Materials

The following supporting information can be downloaded at https://www.mdpi.com/article/10.3390/ijerph20054486/s1: Table S1: Descriptive analysis on socio-demographic information. Table S2: Descriptive analysis on information on health effects induced by environmental pollution. * = the percentage values were calculated excluding the answers “I don’t know” and “I don’t use this information source”. Table S3: Descriptive analysis on information on risk perception of environmental pollution. * = the percentage values were calculated excluding the answer “I don’t know”. Table S4: Descriptive analysis on information on importance of institutional and non-institutional subjects to control the risk due to environmental pollution. * = the percentage values were calculated excluding the answer “I don’t know”. Table S5: Descriptive analysis on information on perceived importance of pro-environmental behaviours. * = the percentage values were calculated excluding the answer “I don’t know”. Table S6: Descriptive analysis on information on adoption of pro-environmental behaviours. * = the percentage values were calculated excluding the answer “I don’t know”. Table S7: Descriptive analysis on barriers to adoption of pro-environmental behaviours. Table S8: Descriptive analysis on motivations to adopt pro-environmental behaviours. * = the percentage values were calculated excluding the answer “I don’t know”.
