# Supplementary material for: The Environmental Health Literacy of Italian General Population: The SPeRA Cross-Sectional Study
Source: Int J Environ Res Public Health. 2023 Mar 2;20(5):4486. doi: 10.3390/ijerph20054486 (PMC10002404; doi:10.3390/ijerph20054486)
Supplement: Supplementary file 1 [file ijerph-20-04486-s001.zip › ijerph-2206465-supplementary.docx]

**Table S1.** Descriptive analysis on socio-demographic information.

| Item | Answer | % | *n* |
| --- | --- | --- | --- |
| Gender | Male | 28.13% | 189 |
|  | Female | 71.13% | 478 |
|  | Not binary | 0.74% | 5 |
| Nationality | Italian | 97.99% | 634 |
|  | Not Italian | 2.01% | 13 |
| Town of residence | Very small town | 23.30% | 144 |
|  | Small town (≤ 50,000 inhabitants) | 40.13% | 248 |
|  | Medium town (50,001 – 250,000 inhabitants) | 21.04% | 130 |
|  | Big town (> 250,000 inhabitants) | 15.53% | 96 |
| Education level | Middle school diploma or less | 11.71% | 72 |
|  | High school diploma | 43.58% | 268 |
|  | Bachelor degree | 15.28% | 94 |
|  | Master degree | 22.11% | 136 |
|  | Doctoral degree | 7.32% | 45 |
| Children | No | 39.74% | 242 |
|  | Yes | 60.26% | 367 |
| Employment status | Worker | 68.60% | 415 |
|  | Unemployed | 10.74% | 65 |
|  | Student | 3.97% | 24 |
|  | Retired | 13.72% | 83 |
|  | Working student | 2.98% | 18 |

**Table S2.** Descriptive analysis on information on health effects induced by environmental pollution. *= the percentage values were calculated excluding the answers “I don’t know” and “I don’t use this information source”.

| Item | Answer | % | *n* |
| --- | --- | --- | --- |
| Opinion on information about health effects induced by environmental pollution | True and complete | 15.10% | 82 |
|  | True but incomplete | 73.66% | 400 |
|  | Not true and incomplete | 11.23% | 61 |
|  | I don't know | -* | 48 |
| How often do you verify information about health effects due to environmental pollution before believing or disclosing it? | Never | 6.32% | 35 |
|  | Sporadically | 15.52% | 86 |
|  | Sometimes | 22.74% | 126 |
|  | Often | 34.84% | 193 |
|  | Always | 20.58% | 114 |
| Main information source about health effects induced by environmental pollution | TV | 42.52% | 256 |
|  | Radio | 7.14% | 43 |
|  | Internet | 70.43% | 424 |
|  | Social Media | 20.43% | 123 |
|  | Newspapers and magazines | 24.09% | 145 |
|  | Friends and family members | 3.49% | 21 |
|  | Government and Universities | 10.96% | 66 |
|  | Medical doctors | 10.47% | 63 |
| Reliability of TV as information source | Not reliable | 6.28% | 30 |
|  | Partially reliable | 53.14% | 254 |
|  | Moderately reliable | 33.26% | 159 |
|  | Always reliable | 7.32% | 35 |
|  | I don’t use this information source | -* | 91 |
| Reliability of radio as information source | Not reliable | 4.69% | 16 |
|  | Partially reliable | 46.92% | 160 |
|  | Moderately reliable | 40.18% | 137 |
|  | Always reliable | 8.21% | 28 |
|  | I don’t use this information source | -* | 225 |
| Reliability of internet as information source | Not reliable | 4.58% | 25 |
|  | Partially reliable | 53.11% | 290 |
|  | Moderately reliable | 36.08% | 197 |
|  | Always reliable | 6.23% | 34 |
|  | I don’t use this information source | -* | 20 |
| Reliability of social media as information source | Not reliable | 28.38% | 126 |
|  | Partially reliable | 55.86% | 248 |
|  | Moderately reliable | 14.64% | 65 |
|  | Always reliable | 1.13% | 5 |
|  | I don’t use this information source | -* | 122 |
| Reliability of newspapers and magazines as information sources | Not reliable | 3.79% | 17 |
|  | Partially reliable | 39.51% | 177 |
|  | Moderately reliable | 46.88% | 210 |
|  | Always reliable | 9.82% | 44 |
|  | I don’t use this information source | -* | 120 |
| Reliability of friends and family members as information sources | Not reliable | 23.28% | 88 |
|  | Partially reliable | 55.03% | 208 |
|  | Moderately reliable | 16.40% | 62 |
|  | Always reliable | 5.29% | 20 |
|  | I don’t use this information source | -* | 185 |
| Reliability of Government and Universities as information sources | Not reliable | 5.30% | 22 |
|  | Partially reliable | 31.08% | 129 |
|  | Moderately reliable | 39.28% | 163 |
|  | Always reliable | 24.34% | 101 |
|  | I don’t use this information source | -* | 149 |
| Reliability of medical doctors as information sources | Not reliable | 2.34% | 10 |
|  | Partially reliable | 15.65% | 67 |
|  | Moderately reliable | 39.95% | 171 |
|  | Always reliable | 42.06% | 180 |
|  | I don’t use this information source | -* | 137 |

**Table S3.** Descriptive analysis on information on risk perception of environmental pollution. *= the percentage values were calculated excluding the answers “I don’t know”.

| Item | Answer | % | *n* |
| --- | --- | --- | --- |
| Self-perceived knowledge about health effects due to environmental pollution | Sufficient | 28.83% | 158 |
|  | Incomplete | 61.86% | 339 |
|  | Insufficient | 9.31% | 51 |
| How often do you feel exposed to environmental pollution? | Never | 0.74% | 4 |
|  | Sporadically | 5.81% | 31 |
|  | Sometimes | 18.35% | 98 |
|  | Often | 45.32% | 242 |
|  | Always | 29.78% | 159 |
| What is the percentage of diseases due to environmental pollution in the world? | 0-20% | 7.89% | 37 |
|  | 21-40% | 29.00% | 136 |
|  | 41-60% | 38.17% | 179 |
|  | >60% | 24.95% | 117 |
|  | I don’t know | -* | 48 |
| Environmental pollution induces | No health effects | 0% | 0 |
|  | Short-term health effects | 5.86% | 31 |
|  | Long-term health effects | 24.01% | 127 |
|  | Short-term and long-term health effects | 70.13% | 371 |
| Environmental pollution mainly induces health effects on | No-one | 0% | 0 |
|  | New-borns | 15.73% | 81 |
|  | Children | 36.12% | 186 |
|  | Adults | 17.67% | 91 |
|  | Elders | 23.88% | 123 |
|  | All people in the same way | 53.01% | 273 |
| Role of environmental pollution in the development of cancers | Not important | 0% | 0 |
|  | Little important | 0.20% | 1 |
|  | Moderately important | 13.95% | 71 |
|  | Very important | 47.74% | 243 |
|  | Extremely important | 38.11% | 194 |
|  | I don’t know | -* | 12 |
| Role of environmental pollution in the development of cardiovascular diseases | Not important | 0.21% | 1 |
|  | Little important | 10.53% | 50 |
|  | Moderately important | 35.58% | 169 |
|  | Very important | 36.42% | 173 |
|  | Extremely important | 17.26% | 82 |
|  | I don’t know | -* | 46 |
| Role of environmental pollution in the development of neurological diseases and dementia | Not important | 3.02% | 13 |
|  | Little important | 19.26% | 83 |
|  | Moderately important | 34.57% | 149 |
|  | Very important | 29.47% | 127 |
|  | Extremely important | 13.67% | 59 |
|  | I don’t know | -* | 90 |
| Role of environmental pollution in the development of congenital and neonatal malformations | Not important | 2.11% | 10 |
|  | Little important | 5.50% | 26 |
|  | Moderately important | 20.30% | 96 |
|  | Very important | 40.80% | 193 |
|  | Extremely important | 31.29% | 148 |
|  | I don’t know | -* | 48 |
| Role of environmental pollution in the development of infectious diseases | Not important | 5.17% | 23 |
|  | Little important | 14.61% | 65 |
|  | Moderately important | 32.13% | 143 |
|  | Very important | 33.26% | 148 |
|  | Extremely important | 14.83% | 66 |
|  | I don’t know | -* | 76 |
| Role of environmental pollution in the development of respiratory diseases | Not important | 0% | 0 |
|  | Little important | 0.78% | 4 |
|  | Moderately important | 7.20% | 37 |
|  | Very important | 33.07% | 170 |
|  | Extremely important | 58.95% | 303 |
|  | I don’t know | -* | 7 |
| Role of environmental pollution in the development of psychiatric disorders | Not important | 11.76% | 46 |
|  | Little important | 28.39% | 111 |
|  | Moderately important | 34.02% | 133 |
|  | Very important | 19.44% | 76 |
|  | Extremely important | 6.39% | 25 |
|  | I don’t know | -* | 130 |
| Role of environmental pollution in the development of gastrointestinal diseases | Not important | 1.54% | 7 |
|  | Little important | 13.63% | 62 |
|  | Moderately important | 40.22% | 183 |
|  | Very important | 29.01% | 132 |
|  | Extremely important | 15.60% | 71 |
|  | I don’t know | -* | 66 |
| Role of environmental pollution in the development of sense organ disorders | Not important | 1.09% | 5 |
|  | Little important | 10.04% | 46 |
|  | Moderately important | 33.62% | 154 |
|  | Very important | 34.72% | 159 |
|  | Extremely important | 20.52% | 94 |
|  | I don’t know | -* | 63 |
| Role of climate change in negatively affecting human health | Not important | 1.20% | 6 |
|  | Little important | 10.44% | 52 |
|  | Moderately important | 15.66% | 78 |
|  | Very important | 41.37% | 206 |
|  | Extremely important | 31.33% | 156 |
|  | I don’t know | -* | 8 |
| Role of vehicular traffic in negatively affecting human health | Not important | 0.20% | 1 |
|  | Little important | 3.96% | 20 |
|  | Moderately important | 9.11% | 46 |
|  | Very important | 40.79% | 206 |
|  | Extremely important | 45.94% | 232 |
|  | I don’t know | -* | 1 |
| Role of nuclear power plants in negatively affecting human health | Not important | 5.59% | 26 |
|  | Little important | 20.22% | 94 |
|  | Moderately important | 15.48% | 72 |
|  | Very important | 23.87% | 111 |
|  | Extremely important | 34.84% | 162 |
|  | I don’t know | -* | 41 |
| Rome of home heating in negatively affecting human health | Not important | 3.12% | 15 |
|  | Little important | 15.80% | 76 |
|  | Moderately important | 27.02% | 130 |
|  | Very important | 40.12% | 193 |
|  | Extremely important | 13.93% | 67 |
|  | I don’t know | -* | 25 |
| Role of industrial accidents in negatively affecting human health | Not important | 1.61% | 8 |
|  | Little important | 10.08% | 50 |
|  | Moderately important | 12.70% | 63 |
|  | Very important | 34.68% | 172 |
|  | Extremely important | 40.93% | 203 |
|  | I don’t know | -* | 10 |
| Role of radio/TV transmitters and cell phones in negatively affecting human health | Not important | 4.31% | 20 |
|  | Little important | 21.12% | 98 |
|  | Moderately important | 22.63% | 105 |
|  | Very important | 34.70% | 161 |
|  | Extremely important | 17.24% | 80 |
|  | I don’t know | -* | 42 |
| Role of genetically modified food in negatively affecting human health | Not important | 10.51% | 47 |
|  | Little important | 15.88% | 71 |
|  | Moderately important | 13.20% | 59 |
|  | Very important | 30.87% | 138 |
|  | Extremely important | 29.53% | 132 |
|  | I don’t know | -* | 59 |
| Role of ozone hole in negatively affecting human health | Not important | 1.25% | 6 |
|  | Little important | 9.77% | 47 |
|  | Moderately important | 14.14% | 68 |
|  | Very important | 38.25% | 184 |
|  | Extremely important | 36.59% | 176 |
|  | I don’t know | -* | 25 |
| Role of industrial pollution in negatively affecting human health | Not important | 0% | 0 |
|  | Little important | 1.39% | 7 |
|  | Moderately important | 6.18% | 31 |
|  | Very important | 35.26% | 177 |
|  | Extremely important | 57.17% | 287 |
|  | I don’t know | -* | 4 |
| Role of chemicals in food and drinking water in negatively affecting human health | Not important | 0.60% | 3 |
|  | Little important | 4.59% | 23 |
|  | Moderately important | 6.99% | 35 |
|  | Very important | 28.94% | 145 |
|  | Extremely important | 58.88% | 295 |
|  | I don’t know | -* | 5 |
| Role of outdoor air quality in negatively affecting human health | Not important | 4.26% | 21 |
|  | Little important | 11.36% | 56 |
|  | Moderately important | 19.47% | 96 |
|  | Very important | 37.73% | 186 |
|  | Extremely important | 27.18% | 134 |
|  | I don’t know | -* | 13 |
| Role of indoor air quality in negatively affecting human health | Not important | 1.64% | 8 |
|  | Little important | 7.98% | 39 |
|  | Moderately important | 13.91% | 68 |
|  | Very important | 42.94% | 210 |
|  | Extremely important | 33.54% | 164 |
|  | I don’t know | -* | 17 |
| Role of groundwater pollution in negatively affecting human health | Not important | 0.20% | 1 |
|  | Little important | 2.40% | 12 |
|  | Moderately important | 5.39% | 27 |
|  | Very important | 35.13% | 176 |
|  | Extremely important | 56.89% | 285 |
|  | I don’t know | -* | 5 |
| Role of traffic noise in negatively affecting human health | Not important | 3.71% | 18 |
|  | Little important | 21.24% | 103 |
|  | Moderately important | 26.39% | 128 |
|  | Very important | 34.85% | 169 |
|  | Extremely important | 13.81% | 67 |
|  | I don’t know | -* | 21 |
| Role of landfills in negatively affecting human health | Not important | 0.83% | 4 |
|  | Little important | 11.62% | 56 |
|  | Moderately important | 16.39% | 79 |
|  | Very important | 34.44% | 166 |
|  | Extremely important | 36.72% | 177 |
|  | I don’t know | -* | 24 |
| Role of incinerators in negatively affecting human health | Not important | 2.29% | 11 |
|  | Little important | 17.92% | 86 |
|  | Moderately important | 17.92% | 86 |
|  | Very important | 31.25% | 150 |
|  | Extremely important | 30.63% | 147 |
|  | I don’t know | -* | 26 |
| Role of surface water pollution in negatively affecting human health | Not important | 0% | 0 |
|  | Little important | 2.98% | 15 |
|  | Moderately important | 6.16% | 31 |
|  | Very important | 32.80% | 165 |
|  | Extremely important | 58.05% | 292 |
|  | I don’t know | -* | 3 |
| Role of biological contamination of food/water in negatively affecting human health | Not important | 1.93% | 9 |
|  | Little important | 12.88% | 60 |
|  | Moderately important | 12.23% | 57 |
|  | Very important | 33.05% | 154 |
|  | Extremely important | 39.91% | 186 |
|  | I don’t know | -* | 40 |
| Role of sunlight exposure without sunscreen in negatively affecting human health | Not important | 1.24% | 6 |
|  | Little important | 9.71% | 47 |
|  | Moderately important | 16.32% | 79 |
|  | Very important | 42.98% | 208 |
|  | Extremely important | 29.75% | 144 |
|  | I don’t know | -* | 5 |
| Role of the use of wood or pellet stoves in negatively affecting human health | Not important | 13.02% | 56 |
|  | Little important | 43.02% | 185 |
|  | Moderately important | 21.63% | 93 |
|  | Very important | 19.07% | 82 |
|  | Extremely important | 3.26% | 14 |
|  | I don’t know | -* | 59 |
| Role of the abuse of household chemicals in negatively affecting human health | Not important | 0.62% | 3 |
|  | Little important | 9.90% | 48 |
|  | Moderately important | 20.41% | 99 |
|  | Very important | 41.24% | 200 |
|  | Extremely important | 27.83% | 135 |
|  | I don’t know | -* | 4 |
| Role of improper food storage in negatively affecting human health | Not important | 4.15% | 19 |
|  | Little important | 27.07% | 124 |
|  | Moderately important | 20.96% | 96 |
|  | Very important | 34.72% | 159 |
|  | Extremely important | 13.10% | 60 |
|  | I don’t know | -* | 31 |
| Role of cigarette smoke in negatively affecting human health | Not important | 0% | 0 |
|  | Little important | 2.88% | 14 |
|  | Moderately important | 4.94% | 24 |
|  | Very important | 38.27% | 186 |
|  | Extremely important | 53.91% | 262 |
|  | I don’t know | -* | 3 |

**Table S4.** Descriptive analysis on information on importance of institutional and not-institutional subjects to control the risk due to environmental pollution. *= the percentage values were calculated excluding the answers “I don’t know”.

| Item | Answer | % | *n* |
| --- | --- | --- | --- |
| Is environmental pollution controllable? | Absolutely not | 15.28% | 70 |
|  | Partially | 41.27% | 189 |
|  | Moderately | 24.24% | 111 |
|  | Abundantly | 16.59% | 76 |
|  | Extremely | 2.62% | 12 |
|  | I don’t know | -* | 13 |
| Is the human health risk due to environmental pollution controllable? | Absolutely not | 24.78% | 113 |
|  | Partially | 40.57% | 185 |
|  | Moderately | 19.52% | 89 |
|  | Abundantly | 12.94% | 59 |
|  | Extremely | 2.19% | 10 |
|  | I don’t know | -* | 15 |
| Is the health risk due to environmental pollution being resolved through governmental and institutional actions? | Absolutely not | 58.45% | 256 |
|  | Partially | 36.30% | 159 |
|  | Moderately | 4.11% | 18 |
|  | Abundantly | 0.91% | 4 |
|  | Extremely | 0.23% | 1 |
|  | I don’t know | -* | 33 |
| Is the health risk due to environmental pollution being resolved through public awareness? | Absolutely not | 41.03% | 183 |
|  | Partially | 49.33% | 220 |
|  | Moderately | 5.16% | 23 |
|  | Abundantly | 4.04% | 18 |
|  | Extremely | 0.45% | 2 |
|  | I don’t know | -* | 25 |
| Importance of medical doctors in controlling the health risk due to environmental pollution | Not important | 3.31% | 15 |
|  | Little important | 9.27% | 42 |
|  | Moderately important | 20.75% | 94 |
|  | Very important | 43.49% | 197 |
|  | Extremely important | 23.17% | 105 |
|  | I don’t know | -* | 11 |
| Importance of the Government in controlling the health risk due to environmental pollution | Not important | 2.64% | 12 |
|  | Little important | 4.63% | 21 |
|  | Moderately important | 7.49% | 34 |
|  | Very important | 36.78% | 167 |
|  | Extremely important | 48.46% | 220 |
|  | I don’t know | -* | 10 |
| Importance of law enforcement agency in controlling the health risk due to environmental pollution | Not important | 11.33% | 51 |
|  | Little important | 20.44% | 92 |
|  | Moderately important | 31.33% | 141 |
|  | Very important | 27.33% | 123 |
|  | Extremely important | 9.56% | 43 |
|  | I don’t know | -* | 14 |
| Importance of citizens in controlling the health risk due to environmental pollution | Not important | 0.44% | 2 |
|  | Little important | 3.06% | 14 |
|  | Moderately important | 8.08% | 37 |
|  | Very important | 30.13% | 138 |
|  | Extremely important | 58.30% | 267 |
|  | I don’t know | -* | 6 |
| Importance of non-governmental organisations in controlling the health risk due to environmental pollution | Not important | 7.58% | 30 |
|  | Little important | 11.11% | 44 |
|  | Moderately important | 28.03% | 111 |
|  | Very important | 32.32% | 128 |
|  | Extremely important | 20.96% | 83 |
|  | I don’t know | -* | 68 |
| Importance of Environmental Ministry in controlling the health risk due to environmental pollution | Not important | 3.30% | 15 |
|  | Little important | 3.52% | 16 |
|  | Moderately important | 9.89% | 45 |
|  | Very important | 30.55% | 139 |
|  | Extremely important | 52.75% | 240 |
|  | I don’t know | -* | 9 |
| Importance of Environmental Protection Agencies in controlling the health risk due to environmental pollution | Not important | 1.33% | 6 |
|  | Little important | 4.20% | 19 |
|  | Moderately important | 12.17% | 55 |
|  | Very important | 32.30% | 146 |
|  | Extremely important | 50.00% | 226 |
|  | I don’t know | -* | 12 |

**Table S5.** Descriptive analysis on information on perceived importance of pro-environmental behaviours. *= the percentage values were calculated excluding the answers “I don’t know”.

| Item | Answer | % | *n* |
| --- | --- | --- | --- |
| Importance of separate waste collection to reduce environmental pollution | Not important | 0.89% | 4 |
|  | Little important | 0.89% | 4 |
|  | Moderately important | 7.32% | 33 |
|  | Very important | 31.93% | 144 |
|  | Extremely important | 58.98% | 266 |
|  | I don’t know | -* | 2 |
| Importance of reducing alcohol consumption to reduce environmental pollution | Not important | 21.74% | 85 |
|  | Little important | 27.62% | 108 |
|  | Moderately important | 23.02% | 90 |
|  | Very important | 15.86% | 62 |
|  | Extremely important | 11.76% | 46 |
|  | I don’t know | -* | 62 |
| Importance of using low-impact products to reduce environmental pollution | Not important | 0.22% | 1 |
|  | Little important | 0.44% | 2 |
|  | Moderately important | 8.65% | 39 |
|  | Very important | 34.15% | 154 |
|  | Extremely important | 56.54% | 255 |
|  | I don’t know | -* | 2 |
| Importance of reducing energy consumption to reduce environmental pollution | Not important | 0.44% | 2 |
|  | Little important | 1.55% | 7 |
|  | Moderately important | 9.76% | 44 |
|  | Very important | 40.35% | 182 |
|  | Extremely important | 47.89% | 216 |
|  | I don’t know | -* | 2 |
| Importance of online shopping to reduce environmental pollution | Not important | 37.66% | 148 |
|  | Little important | 30.79% | 121 |
|  | Moderately important | 21.37% | 84 |
|  | Very important | 6.87% | 27 |
|  | Extremely important | 3.31% | 13 |
|  | I don’t know | -* | 60 |
| Importance of sustainable tourism to reduce environmental pollution | Not important | 2.75% | 12 |
|  | Little important | 5.49% | 24 |
|  | Moderately important | 29.06% | 127 |
|  | Very important | 38.44% | 168 |
|  | Extremely important | 24.26% | 106 |
|  | I don’t know | -* | 16 |
| Importance of reducing home heating during winter to reduce environmental pollution | Not important | 1.58% | 7 |
|  | Little important | 4.52% | 20 |
|  | Moderately important | 31.00% | 137 |
|  | Very important | 41.63% | 184 |
|  | Extremely important | 21.27% | 94 |
|  | I don’t know | -* | 11 |
| Importance of reducing air conditioning during summer to reduce environmental pollution | Not important | 1.58% | 7 |
|  | Little important | 4.74% | 21 |
|  | Moderately important | 24.60% | 109 |
|  | Very important | 41.31% | 183 |
|  | Extremely important | 27.76% | 123 |
|  | I don’t know | -* | 10 |
| Importance of planting trees to reduce environmental pollution | Not important | 2.51% | 11 |
|  | Little important | 7.52% | 33 |
|  | Moderately important | 27.11% | 119 |
|  | Very important | 31.89% | 140 |
|  | Extremely important | 30.98% | 136 |
|  | I don’t know | -* | 14 |
| Importance of purchasing zero-mile products to reduce environmental pollution | Not important | 1.80% | 8 |
|  | Little important | 4.28% | 19 |
|  | Moderately important | 19.37% | 86 |
|  | Very important | 40.76% | 181 |
|  | Extremely important | 33.78% | 150 |
|  | I don’t know | -* | 9 |
| Importance of reducing meat consumption to reduce environmental pollution | Not important | 3.89% | 17 |
|  | Little important | 10.76% | 47 |
|  | Moderately important | 28.60% | 125 |
|  | Very important | 30.21% | 132 |
|  | Extremely important | 26.54% | 116 |
|  | I don’t know | -* | 16 |
| Importance of doing physical activity to reduce environmental pollution | Not important | 12.29% | 52 |
|  | Little important | 6.86% | 29 |
|  | Moderately important | 14.42% | 61 |
|  | Very important | 35.93% | 152 |
|  | Extremely important | 30.50% | 129 |
|  | I don’t know | -* | 30 |
| Importance of purchasing ecolabel products to reduce environmental pollution | Not important | 3.90% | 13 |
|  | Little important | 8.11% | 27 |
|  | Moderately important | 35.44% | 118 |
|  | Very important | 34.23% | 114 |
|  | Extremely important | 18.32% | 61 |
|  | I don’t know | -* | 120 |
| Importance of using green fuels to reduce environmental pollution | Not important | 1.12% | 5 |
|  | Little important | 2.25% | 10 |
|  | Moderately important | 12.81% | 57 |
|  | Very important | 44.72% | 199 |
|  | Extremely important | 39.10% | 174 |
|  | I don’t know | -* | 8 |
| Importance of using public transports to reduce environmental pollution | Not important | 0.68% | 3 |
|  | Little important | 2.93% | 13 |
|  | Moderately important | 22.97% | 102 |
|  | Very important | 46.85% | 208 |
|  | Extremely important | 26.58% | 118 |
|  | I don’t know | -* | 9 |
| Importance of using sustainable transports to reduce environmental pollution (e.g. bicycles, car-sharing, electric transport vehicles) | Not important | 0.89% | 4 |
|  | Little important | 2.22% | 10 |
|  | Moderately important | 15.78% | 71 |
|  | Very important | 40.22% | 181 |
|  | Extremely important | 40.89% | 184 |
|  | I don’t know | -* | 3 |

**Table S6.** Descriptive analysis on information on adoption of pro-environmental behaviours. *= the percentage values were calculated excluding the answers “I don’t know”.

| Item | Answer | % | *n* |
| --- | --- | --- | --- |
| How often do you separate waste? | Never | 0.22% | 1 |
|  | Sporadically | 0.45% | 2 |
|  | Sometimes | 3.36% | 15 |
|  | Often | 11.86% | 53 |
|  | Always | 84.12% | 376 |
|  | I don’t know | -* | 0 |
| How often do you reduce alcohol consumption? | Never | 5.75% | 25 |
|  | Sporadically | 6.21% | 27 |
|  | Sometimes | 21.15% | 92 |
|  | Often | 25.06% | 109 |
|  | Always | 41.84% | 182 |
|  | I don’t know | -* | 12 |
| How often do you use low-impact products? | Never | 0.67% | 3 |
|  | Sporadically | 6.95% | 31 |
|  | Sometimes | 15.25% | 68 |
|  | Often | 43.95% | 196 |
|  | Always | 33.18% | 148 |
|  | I don’t know | -* | 1 |
| How often do you reduce energy consumption? | Never | 0.22% | 1 |
|  | Sporadically | 4.94% | 22 |
|  | Sometimes | 18.20% | 81 |
|  | Often | 40.90% | 182 |
|  | Always | 35.73% | 159 |
|  | I don’t know | -* | 2 |
| How often do you shop online? | Never | 5.90% | 26 |
|  | Sporadically | 28.57% | 126 |
|  | Sometimes | 31.07% | 137 |
|  | Often | 31.29% | 138 |
|  | Always | 3.17% | 14 |
|  | I don’t know | -* | 6 |
| How often do you do sustainable tourism? | Never | 8.98% | 37 |
|  | Sporadically | 16.26% | 67 |
|  | Sometimes | 32.28% | 133 |
|  | Often | 29.13% | 120 |
|  | Always | 13.35% | 55 |
|  | I don’t know | -* | 35 |
| How often do you reduce home heating during winter? | Never | 4.06% | 18 |
|  | Sporadically | 12.42% | 55 |
|  | Sometimes | 39.95% | 177 |
|  | Often | 29.57% | 131 |
|  | Always | 14.00% | 62 |
|  | I don’t know | -* | 3 |
| How often do you reduce air conditioning during summer? | Never | 2.75% | 12 |
|  | Sporadically | 9.17% | 40 |
|  | Sometimes | 21.56% | 94 |
|  | Often | 26.83% | 117 |
|  | Always | 39.68% | 173 |
|  | I don’t know | -* | 10 |
| How often do you plant trees? | Never | 9.42% | 41 |
|  | Sporadically | 14.48% | 63 |
|  | Sometimes | 19.54% | 85 |
|  | Often | 27.82% | 121 |
|  | Always | 28.74% | 125 |
|  | I don’t know | -* | 11 |
| How often do you purchase zero-mile products? | Never | 3.19% | 14 |
|  | Sporadically | 23.01% | 101 |
|  | Sometimes | 35.31% | 155 |
|  | Often | 30.98% | 136 |
|  | Always | 7.52% | 33 |
|  | I don’t know | -* | 7 |
| How often do you reduce meat consumption? | Never | 6.53% | 29 |
|  | Sporadically | 19.37% | 86 |
|  | Sometimes | 33.11% | 147 |
|  | Often | 24.10% | 107 |
|  | Always | 16.89% | 75 |
|  | I don’t know | -* | 2 |
| How often do you do physical activity? | Never | 4.51% | 20 |
|  | Sporadically | 23.70% | 105 |
|  | Sometimes | 31.83% | 141 |
|  | Often | 24.60% | 109 |
|  | Always | 15.35% | 68 |
|  | I don’t know | -* | 3 |
| How often do you purchase ecolabel products? | Never | 13.45% | 46 |
|  | Sporadically | 24.56% | 84 |
|  | Sometimes | 33.04% | 113 |
|  | Often | 23.39% | 80 |
|  | Always | 5.56% | 19 |
|  | I don’t know | -* | 104 |
| How often do you use green fuels? | Never | 15.61% | 64 |
|  | Sporadically | 24.63% | 101 |
|  | Sometimes | 24.88% | 102 |
|  | Often | 21.95% | 90 |
|  | Always | 12.93% | 53 |
|  | I don’t know | -* | 36 |
| How often do you use public transports? | Never | 24.26% | 106 |
|  | Sporadically | 32.49% | 142 |
|  | Sometimes | 13.96% | 61 |
|  | Often | 18.99% | 83 |
|  | Always | 10.30% | 45 |
|  | I don’t know | -* | 9 |
| How often do you use sustainable transports (e.g. bicycles, car-sharing, electric transport vehicles)? | Never | 25.80% | 113 |
|  | Sporadically | 27.40% | 120 |
|  | Sometimes | 16.89% | 74 |
|  | Often | 19.18% | 84 |
|  | Always | 10.73% | 47 |
|  | I don’t know | -* | 8 |

**Table S7.** Descriptive analysis on barriers to adoption of pro-environmental behaviours.

| Item | Answer | % | *n* |
| --- | --- | --- | --- |
| Which are the main barriers to separate waste? | Lack of institutional support | 42.07% | 175 |
|  | Lack of support from family members | 17.55% | 73 |
|  | Lack of time | 8.89% | 37 |
|  | Doubts about its effectiveness | 25.48% | 106 |
|  | Cost | 6.01% | 25 |
| Which are the main barriers to reducing alcohol consumption? | Lack of institutional support | 28.43% | 118 |
|  | Lack of support from family members | 16.14% | 67 |
|  | Lack of time | 6.27% | 26 |
|  | Doubts about its effectiveness | 39.28% | 163 |
|  | Cost | 9.88% | 41 |
| Which are the main barriers to using low-impact products? | Lack of institutional support | 36.96% | 153 |
|  | Lack of support from family members | 8.70% | 36 |
|  | Lack of time | 16.18% | 67 |
|  | Doubts about its effectiveness | 5.80% | 24 |
|  | Cost | 32.37% | 134 |
| Which are the main barriers to reducing energy consumption? | Lack of institutional support | 30.92% | 128 |
|  | Lack of support from family members | 10.87% | 45 |
|  | Lack of time | 7.49% | 31 |
|  | Doubts about its effectiveness | 3.62% | 15 |
|  | Cost | 47.10% | 195 |
| Which are the main barriers to shopping online? | Lack of institutional support | 15.50% | 64 |
|  | Lack of support from family members | 8.72% | 36 |
|  | Lack of time | 22.28% | 92 |
|  | Doubts about its effectiveness | 33.17% | 137 |
|  | Cost | 20.34% | 84 |
| Which are the main barriers to sustainable tourism? | Lack of institutional support | 24.27% | 100 |
|  | Lack of support from family members | 6.55% | 27 |
|  | Lack of time | 18.45% | 76 |
|  | Doubts about its effectiveness | 12.14% | 50 |
|  | Cost | 38.59% | 159 |
| Which are the main barriers to reducing home heating during winter? | Lack of institutional support | 32.12% | 132 |
|  | Lack of support from family members | 33.33% | 137 |
|  | Lack of time | 5.60% | 23 |
|  | Doubts about its effectiveness | 11.44% | 47 |
|  | Cost | 17.52% | 72 |
| Which are the main barriers to reducing air conditioning during summer? | Lack of institutional support | 32.12% | 132 |
|  | Lack of support from family members | 32.60% | 134 |
|  | Lack of time | 6.33% | 26 |
|  | Doubts about its effectiveness | 10.95% | 45 |
|  | Cost | 18.00% | 74 |
| Which are the main barriers to planting trees? | Lack of institutional support | 19.22% | 79 |
|  | Lack of support from family members | 8.52% | 35 |
|  | Lack of time | 47.93% | 197 |
|  | Doubts about its effectiveness | 6.81% | 28 |
|  | Cost | 17.52% | 72 |
| Which are the main barriers to purchasing zero-mile products? | Lack of institutional support | 20.68% | 85 |
|  | Lack of support from family members | 4.87% | 20 |
|  | Lack of time | 26.28% | 108 |
|  | Doubts about its effectiveness | 5.84% | 24 |
|  | Cost | 42.34% | 174 |
| Which are the main barriers to reducing meat consumption? | Lack of institutional support | 20.29% | 84 |
|  | Lack of support from family members | 29.71% | 123 |
|  | Lack of time | 11.35% | 47 |
|  | Doubts about its effectiveness | 23.43% | 97 |
|  | Cost | 15.22% | 63 |
| Which are the main barriers to doing physical activity? | Lack of institutional support | 10.44% | 43 |
|  | Lack of support from family members | 5.58% | 23 |
|  | Lack of time | 72.09% | 297 |
|  | Doubts about its effectiveness | 5.10% | 21 |
|  | Cost | 6.80% | 28 |
| Which are the main barriers to purchasing ecolabel products? | Lack of institutional support | 18.00% | 74 |
|  | Lack of support from family members | 5.60% | 23 |
|  | Lack of time | 11.19% | 46 |
|  | Doubts about its effectiveness | 15.57% | 64 |
|  | Cost | 49.64% | 204 |
| Which are the main barriers to using green fuels? | Lack of institutional support | 39.81% | 164 |
|  | Lack of support from family members | 3.16% | 13 |
|  | Lack of time | 3.64% | 15 |
|  | Doubts about its effectiveness | 6.80% | 28 |
|  | Cost | 46.60% | 192 |
| Which are the main barriers to using public transports? | Lack of institutional support | 46.36% | 191 |
|  | Lack of support from family members | 2.67% | 11 |
|  | Lack of time | 31.55% | 130 |
|  | Doubts about its effectiveness | 8.01% | 33 |
|  | Cost | 11.41% | 47 |
| Which are the main barriers to using sustainable transports (e.g. bicycles, car-sharing, electric transport vehicles)? | Lack of institutional support | 32.77% | 135 |
|  | Lack of support from family members | 4.61% | 19 |
|  | Lack of time | 40.78% | 168 |
|  | Doubts about its effectiveness | 6.07% | 25 |
|  | Cost | 15.78% | 65 |

**Table S8.** Descriptive analysis on motivations to adopt pro-environmental behaviours. *= the percentage values were calculated excluding the answers “I don’t know”.

| Item | Answer | % | *n* |
| --- | --- | --- | --- |
| Importance of economic benefit as motivation to adopt pro-environmental behaviours | Not important | 7.43% | 30 |
|  | Little important | 17.33% | 70 |
|  | Moderately important | 32.18% | 130 |
|  | Very important | 25.99% | 105 |
|  | Extremely important | 17.08% | 69 |
| Importance of approval by other people as motivation to adopt pro-environmental behaviours | Not important | 30.69% | 124 |
|  | Little important | 31.19% | 126 |
|  | Moderately important | 16.09% | 65 |
|  | Very important | 14.60% | 59 |
|  | Extremely important | 7.43% | 30 |
| Importance of protection of the environment as motivation to adopt pro-environmental behaviours | Not important | 0.00% | 0 |
|  | Little important | 0.50% | 2 |
|  | Moderately important | 6.19% | 25 |
|  | Very important | 27.97% | 113 |
|  | Extremely important | 65.35% | 264 |
| Importance of protection of oneself health as motivation to adopt pro-environmental behaviours | Not important | 0.00% | 0 |
|  | Little important | 0.50% | 2 |
|  | Moderately important | 3.71% | 15 |
|  | Very important | 23.76% | 96 |
|  | Extremely important | 72.03% | 291 |
| Importance of protection of other people health as motivation to adopt pro-environmental behaviours | Not important | 0.00% | 0 |
|  | Little important | 0.00% | 0 |
|  | Moderately important | 8.17% | 33 |
|  | Very important | 27.48% | 111 |
|  | Extremely important | 64.36% | 260 |
